# Contactin-1/F3 Regulates Neuronal Migration and Morphogenesis Through Modulating RhoA Activity

**DOI:** 10.3389/fnmol.2018.00422

**Published:** 2018-11-20

**Authors:** Yi-An Chen, I-Ling Lu, Jin-Wu Tsai

**Affiliations:** ^1^Institute of Brain Science, National Yang-Ming University, Taipei, Taiwan; ^2^Brain Research Center, National Yang-Ming University, Taipei, Taiwan; ^3^Biophotonics and Molecular Imaging Research Center, National Yang-Ming University, Taipei, Taiwan

**Keywords:** contactin-1, Cntn1, contactin, cortical development, neuronal migration, cell adhesion, RhoA, *in utero* electroporation

## Abstract

During neocortical development, newborn neurons migrate along radial fibers from the germinal ventricular zone (VZ) toward the cortical plate (CP) to populate the cerebral cortex. This radial migration requires adhesion activities between neurons and radial fibers; however, past research has identified only a limited number of adhesion molecules involved in this process. Contactin-1/F3 (Cntn1), a cell adhesion molecule expressed in the developing nervous system is essential for many key developmental events including neural cell adhesion, neurite outgrowth, axon guidance and myelination. However, the potential role of Cntn1 in neuronal migration during cortical development has not been investigated. Here we used *in utero* electroporation to introduce short hairpin RNA (shRNA) to knock down (KD) Cntn1 in neural stem cells *in vivo*. We found that Cntn1 KD led to a delay in neuronal migration. The arrested cells presented abnormal morphology in their leading process and more multipolar cells were observed in the deep layers of the brain, suggestive of dysregulation in process formation. Intriguingly, Cntn1 KD also resulted in upregulation of RhoA, a negative regulator for neuronal migration. Interference of RhoA by expression of the dominant-negative RhoA^N19^ partially rescued the neuronal migration defects caused by Cntn1 KD. Our results showed that Cntn1 is a novel adhesion protein that is essential for neuronal migration and regulates process formation of newborn cortical neurons through modulating RhoA signaling pathway.

## Introduction

In the cerebral cortex, the highly organized pyramidal neurons originate directly or indirectly from RGCs located in the deep proliferative germinal region, known as the VZ (Kriegstein and Noctor, 2004; Molyneaux et al., 2007). The newborn neurons migrate along radial fiber of RGCs to the CP. Perturbations in neuronal migration can lead to severe neurodevelopmental and cognitive disorders such as lissencephaly, cortical band heterotopia, and double cortex syndrome (Tsai et al., 2005, 2007; Vallee and Tsai, 2006; Ayala et al., 2007; Kerjan and Gleeson, 2007; Liu, 2011; Moon and Wynshaw-Boris, 2013; Guerrini and Dobyns, 2014; Tsai et al., 2016).

Neuronal migration involves many cellular activities, such as neurite guidance, cell adhesion, force generation, and transport of organelles (Vallee et al., 2009; He et al., 2010; Solecki, 2012; Jheng et al., 2018). Although progress has been made in identification of adhesion molecules between migrating granule cells and radial glia during cerebellar development, cell adhesion molecules involved in radial migration of cortical neurons are relatively less studied (Solecki, 2012). To date, a limited number of molecules have been identified to be involved in neuronal migration in the developing cortex, such as *N*-cadherin, integrins, and connexins (Kawauchi, 2015). Suppression of *N*-cadherin perturbs the attachment of migrating neurons to the radial glial fibers (Kawauchi et al., 2010). The integrin heterodimers are involved in Reelin-dependent neuronal positioning (Sekine et al., 2012). The treatment with antibodies against β1-integrin also suppresses radial glial fiber-dependent neuronal migration *in vitro* (Anton et al., 1999). In addition, the gap junction subunits connexin 26 (Cx26) and connexin 43 (Cx43) are found to be expressed at the contact points between radial fibers and migrating neurons. Cx26 and Cx43 may provide dynamic adhesive contacts that interact with the internal cytoskeleton (Elias et al., 2007).

Contactins, a subgroup of adhesion molecules belonging to the immunoglobulin superfamily, are expressed primarily in the nervous system. The contactin family is comprised of 6 members: Cntn1 (F3), Cntn2 (TAG-1/TAX-1/axonin), Cntn3 (PANG/BIG-1), Cntn4 (BIG-2), Cntn5 (NB-2), and Cntn6 (NB-3) (Shimoda and Watanabe, 2009). Cntn1 is a GPI-anchored membrane protein with 6 immunoglobulin (Ig)-like domains and 4 fibronectin type III-like domains. Heterophilic interactions between Cntn1 and its various ligands are required in key developmental events such as neural cell adhesion, myelination, neurite growth, axonal elongation and fasciculation (Falk et al., 2002; Karagogeos, 2003). For instance, binding between Cntn1 and Nr-CAM modulates axonal elongation of the cerebellar granule cells and controls sensory axon guidance (Faivre-Sarrailh et al., 1999; Falk et al., 2002). Cntn1 also mediates neuron–glial contacts through its association with extracellular matrix components such as Tenascin-R, Tenascin-C, and the glial surface receptor RPTPβ/Phosphacan. These interactions consequently influence axonal growth and fasciculation (Pesheva et al., 1993; Peles et al., 1995; Xiao et al., 1996; Sakurai et al., 1997; Falk et al., 2002). In addition, Cntn1 organizes axonal subdomains at the node of Ranvier of myelinated fibers in interplay with other Ig-CAMs, such as Caspr/Paranodin at paranodes and the voltage-gated sodium channels in the nodal region (Rios et al., 2000; Boyle et al., 2001). In Cntn1 knockout mice, the granule cell axon guidance and dendritic projections are defective in the cerebellum, leading to serious ataxia (Berglund et al., 1999).

Although early studies focused on the functional roles of Cntn1 in axons, a recent study showed that regulated expression of Cntn1 is critical in maintaining the normal timing of neurogenesis during cortical development (Bizzoca et al., 2012). Overexpression of Cntn1 in the VZ promoted proliferation and expanded the precursor pool at the expense of neurogenesis. In a recent genetic screen using transposon-mediated mutagenesis in the mouse cortex, we also identified Cntn1 as a key factor for cortical development and developmental disorders (Lu et al., 2018). Given the role of Cntn1 in cell adhesion, we postulate that Cntn1 may be involved in neuronal migration; however, this possibility has yet been tested.

In the current study, we aimed to investigate whether Cntn1 may play roles in neuronal migration during corticogenesis. We knocked down Cntn1 expression in the developing cortex acutely by *in utero* electroporation of constructs expressing shRNA in to mouse embryos. We found that Cntn1 KD caused a delay in neuronal migration and defects in multipolar-to-bipolar transition. The Cntn1 KD neurons also exhibited aberrant branched leading processes. In addition, *in vivo* analysis by a fluorescence resonance energy transfer (FRET)-based RhoA probe showed that Cntn1 KD dramatically increased RhoA activity in neural precursors during brain development. Remarkably, expression of a dominant-negative form of RhoA rescued the migration delay caused by Cntn1 KD. Our results suggested that Cntn1 has a novel function in neuronal migration through down regulation of RhoA activity in the developing cortex.

## Materials and Methods

### Animal Model

Timed pregnant ICR mice were used for *in utero* electroporation. The morning of the vaginal plug is embryonic day 0.5 (E0.5), and the first postnatal day (P) is P0. Experiments were done following IACUC guidelines and approved protocols at National Yang-Ming University.

### Constructs

All Cntn1 shRNAs were obtained from the National RNAi Core Facility in Taiwan. The targeting sequences were chosen from a portion of the Cntn1-coding region as follows: 018: CGGCAATCTCTACATCGCAAA; 016: GCCTTCAACAATAAAGGA GAT; 015: GCAGCCAATCAATACCATTTA. These sequences were inserted into pLKO_TRC001 (without GFP) or pLKO-TRC011 (with GFP) vectors (National RNAi Core Facility at Academia Sinica, Taiwan). Plasmids used for *in utero* electroporation were prepared using the MaxiPrep EndoFree Plasmid kit (Qiagen). To fluorescently label cells *in vivo*, shRNA was co-transfected with a pCAG-EGFP vector. The RhoA mutant form construct pEGFP-C1-RhoA^N19^ was a generous gift from Dr. Hsiao-Hui Lee (National Yang-Ming University). We subcloned RhoA^N19^ into a pCIG2-EGFP vector (gift from Dr. Olivier Ayrault, Curie Institute, France), which contains an (cDNA)-IRES-EGFP expression cassette expressed under the control of a CMV-enhancer and a chicken β-actin promoter. Mouse Cntn1 cDNA was derived from MGC clone (Clone ID: BC066864, Transomic Technologies).

### Primary Cortical Neuron Culture and Lentivirus Transduction

Cortical neurons were prepared from E14.5 mouse embryos via papain dissociation system kit (Worthington, Cat. No. LK003153) as described previously (Huettner and Baughman, 1986; Lu et al., 2018). Neurons were cultured in Neural Basal Medium/L-glutamine/B27/penicillin/streptomycin (Cold Spring Harbor Protocols). Lentivirus carrying Cntn1 shRNA or scramble sequence was transduced into primary cortical neurons at DIV 3 and assayed at DIV5. All shRNA reagents and lentivirus particles were purchased from the National RNAi Core Facility at Academia Sinica, Taiwan.

### *In utero* Electroporation

The procedure of *in utero* electroporation was described previously (Liu et al., 2016; Chen et al., 2018; Lu et al., 2018). Electroporation surgery took place at E14.5. Animals were anesthetized with 2 % isoflurane, and abdominally shaved before placed onto a warming pad. The abdominal area was sterilized with 70% alcohol. An incision was made through the skin and abdominal muscle to expose the underlying viscera. The uterine horns were carefully externalized. Each embryo was injected with a solution containing 0.5 μl DNA (shRNA: EGFP = 1:1) at 2 μg/μl concentration into the right lateral ventricle of the developing brain. Electroporation was applied at 50 V, with five 50 ms pulses at 450 ms intervals. After electroporation, the uterine horns were placed back into the abdominal cavity and the incision was closed by suture (5-0, vicryl).

Embryos were harvested 2, 4, and 6 days after electroporation. The pregnant mice were anesthetized using the combination of Avertin and Xylazine. The depth of the anesthesia was tested by pinching the toes and the tail. We used transcardial perfusion by PBS and fixation with 4% Para-formaldehyde (PFA) solution. Cerebral cortices of embryos were immersed in PFA immediately after collection.

### Immunohistochemistry

Cerebral cortices were fixed in PFA overnight and sectioned coronally by Vibratome into 100 μm slices. Slices were washed in PBS and then permeated in PBST solution (0.2% Triton X-100 in PBS) for 15 min. Antigen retrieval was done with citrate buffer (100 mM citrate with 0.1% Triton X-100, pH = 6) in boiling water for 10 min. After cooling and PBS washing, slices were blocked at room temperature with 10% normal goat serum and 5% BSA in PBST solution. Primary antibodies were used with the following concentrations: mouse anti-Cntn1, 1:100 (LSBio); rabbit anti-Cntn1, 1:100 (Proteintech); mouse anti-Tuj1, 1:1000 (Convance); rabbit anti-NeuN, 1:500 (Millipore); rabbit anti-Pax6, 1:300 (BioLegend); rabbit anti-Tbr2, 1:500 (Abcam); mouse anti-RhoA, 1:100 (Santa Cruz). After incubation in primary antibody for two nights, slices were washed in PBS. Following the wash, secondary antibodies (Alexa Fluor, 1:500) were applied for 2 h at room temperature. Finally, the slices were counterstained with 0.5 μg/ml DAPI (Invitrogen) for 1 h. VECTASHIELD Mounting Media was added before sealing the slices. The slices were preserved and kept in a dark place.

### Immunocytochemistry

Cells were fixed with 4% PFA and then permeabilized in PBST solution (0.2% Triton X-100 in PBS) for 15 min. Cells were blocked at room temperature with 10% normal goat serum and 5% BSA in PBS. Primary antibodies were used at the following concentrations: mouse anti-Tuj1, 1:1000 (Convance); mouse anti-Cntn1, 1:1000 (LSBio); rabbit anti-Cntn1, 1:1000 (Proteintech, 13843-1-AP); mouse anti-RhoA, 1:1000 (Santa Cruz). After incubation in primary antibody overnight at 4°C, cells were washed in PBS. Alexa Fluor series (1:500) was then applied for 1 h. Finally, cells were counterstained with DAPI for 30 min at room temperature.

### Microscopy

Slices were imaged under an inverted laser scanning confocal microscope (LSM-700, Zeiss). The excitation wavelengths were 405 nm for DAPI, 488 nm for EGFP, 546 nm for red fluorescence, and 647 nm for infrared.

### Live Cell Imaging in Brain Slices

The brain slice imaging was performed as described previously (Tsai et al., 2005, 2007; Chen et al., 2018). Briefly, slices of coronal sections at 350-μm thickness were prepared using Vibrotome (Leica) 48 h after electroporation. Slices were placed on Millicell-CM inserts (Millipore) in culture medium containing 25% Hanks balanced salt solution, 47% basal MEM, 25% normal horse serum, 1% penicillin/streptomycin/glutamine (GIBCO BRL), and 0.66% glucose. GFP-positive cells were imaged by an inverted fluorescent microscope (Carl Zeiss) with an incubator maintained at 37°C in 5% CO_2_. Time-lapse images were captured at 10-min intervals.

### Fluorescence Resonance Energy Transfer

An inverted laser scanning confocal microscope (LSM-880, Zeiss) was used to record the fluorescence of CFP and YFP. Both fluorescences were excited by 440 and 514 nm light, respectively. The fluorescent expressing (pCAGGS-pRaichu-RhoA plasmid was provided by Michiyuki Matsuda, Kyoto University) was determined by the acceptor photobleaching method of FRET detection. Selective photobleaching of YFP was performed by repeatedly scanning a region of the specimen with the 514 nm laser line set at maximum intensity to photobleach. The pre- and post-bleaching images were analyzed by the ZEN 2012 (blue edition). The efficiency of FRET was calculated by AccPbFRET plugin with ImageJ (Roszik et al., 2008). The efficiency maps were generated by Matlab (MathWorks). The relative intensity is presented as: (post-bleaching of CFP intensity in bleaching area)/(pre-bleaching of CFP intensity in bleaching area).

### Western Blotting

The cell lysates were collected by lysing cells under RIPA buffer (50 mM Tris-HCl, pH 8.0, 150 mM NaCl, 1% NP-40, 0.5% sodium deoxycholate, 0.1% SDS) and mixed with protease inhibitor cocktail (Sigma-Aldrich) and phosphatase inhibitor cocktail (Roche). After removing the cell debris by centrifuging with 15000 rpm for 30 min at 4°C, proteins were quantified by BCA protein assay (Pierce). Next, electrophoresis was performed on 30% acrylamide/bis-acrylamide gradient gel (TOOLS biotechnology), and then transferred to a PVDF membrane (Millipore). After blocking, the following primary antibodies were used: mouse anti-Cntn1 (1:500, LSBio; 1:500, Proteintech, 13843-1-AP), mouse anti-α-Tubulin (1:1000, Proteintech). α-Tubulin was used as an internal control. Primary antibodies were detected through horseradish peroxidase (HRP)-conjugated secondary antibodies listed below: anti-mouse (1:10000, GeneTex), anti-rabbit (1:20000, Sigma-Aldrich). Signals were generated by ECL-Plus reagent (Millipore), and detected under Luminescence/Fluorescence Imaging System LAS-4000 (Fujifilm). Signal quantifications were performed under Image-J based analysis.

## Results

### Neuronal Migration Delay Resulting From Cntn1 KD

**FIGURE 1:**
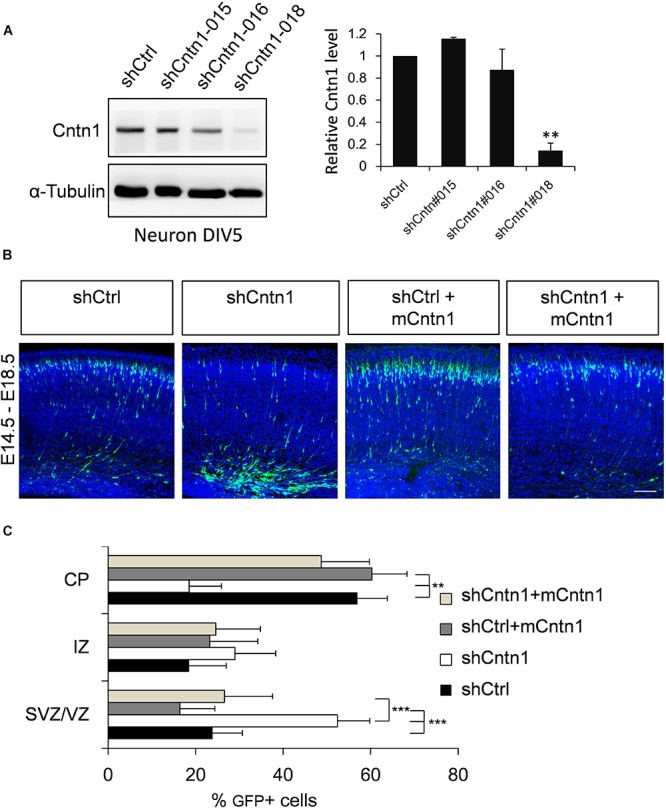
Cntn1 KD impaired radial migration of neurons in the developing mouse neocortex. **(A)** Western blot showing Cntn1 expression in cultured mouse cortical neurons cells 48 h after transfected with shCtrl or 3 different Cntn1 shRNA (left panel) at DIV3. Cntn1 expression in cells transfected with Cntn1 shRNA #018 was markedly lower than that in control cells (right panel). *n* = 3. ^∗∗^*p* < 0.01, one way ANOVA, Bonferroni *post hoc* correction. Data are shown as mean ± *SD*. **(B)** Cell distribution in the developing mouse neocortex 4 days after *in utero* electroporation of shCtrl, shCntn1, and along with mouse Cntn1 (mCntn1) at E14.5. In control brains electroporated with shCtrl, most cells have migrated from the VZ to the CP. In brains electroporated with shCntn1, many cells were restricted to the VZ and IZ. Electoporation of mCntn1 exhibited normal cell distributions. The cell distribution defect by shCntn1 was partially rescued by expression mCntn1. Blue: DAPI. Bar = 100 μm. **(C)** Bar graph of the cell distribution 4 days after electroporation. *n* = 3 animals. ^∗∗^*p* < 0.01, ^∗∗∗^*p* < 0.001, one way ANOVA, Bonferroni *post hoc* correction. Data are shown as mean ± *SD*.

To investigate the role of Cntn1 in brain development, we first examined the effects of Cntn1 knockdown by shRNA in the developing mouse cortex. To select suitable shRNA constructs, cultured cortical neurons were infected with lentiviruses expressing three different shRNA (shCntn1-015, shCntn1-016, and shCntn1-018). We found that shCntn1-018 (referred as shCntn1 hereafter) showed ∼90% downregulation of Cntn1 expression 2 days after transfection (Figure 1A) and thus selected for *in utero* electroporation experiments.

Constructs expressing shCntn1 or control scrambled shRNA (shCtrl) along with EGFP were electroporated into mouse embryos at E14.5, at which time Cntn1 expression emerges (Bizzoca et al., 2012) (Supplementary Figure 1). The distributions of electroporated cells were examined after electroporation in fixed brain slices (Figures 1B,C and Supplementary Figure 2). In the control brain, neural precursor cells progressively migrated from the VZ (Supplementary Figure 2B) and many have reached the CP by day 4 (Figures 1B,C). In contrast, many cells expressing shCntn1 remained in the IZ and VZ (Figures 1B,C and Supplementary Figure 2B). This phenotype was rescued by co-expression of shRNA-resistant mouse Cntn1 in the knockdown cells (Figures 1B,C).

**FIGURE 2:**
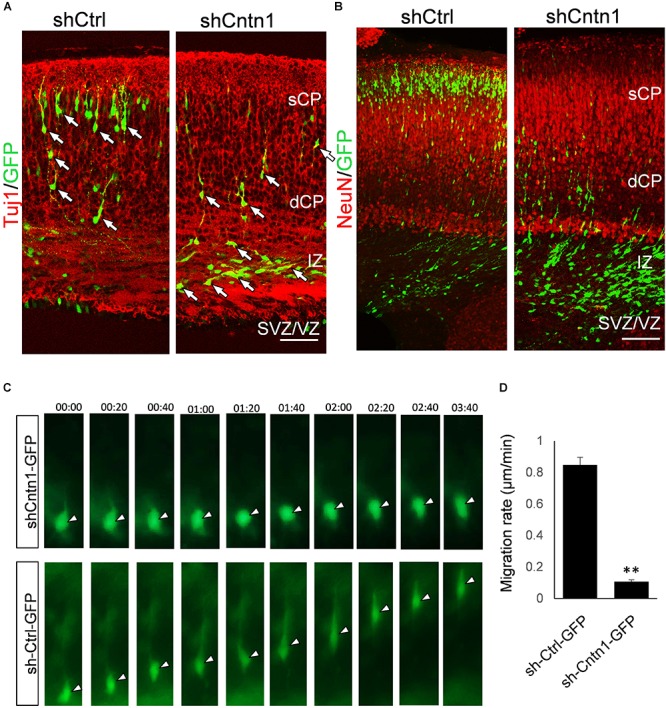
Neuronal lineage and decreased rate of migration in Cntn1-KD cells. **(A)** Immunostaining of neuronal Tuj1 (red) in brain slices electroporated with control or Cntn1 shRNA. The majority of the electroporated GFP+ (green) cells in both control and Cntn1 KD brains were Tuj1+ albeit the difference in cell distribution. Scale bar = 50 μm. **(B)** Most of the GFP+ (green) neurons were still negative to NeuN (red) 4 days after electroporation. Scale bar = 50 μm. **(C)** Live cell imaging of cortical neurons in brain slices. The brains were electroporated with shCntn1 or shCtrl along with GFP at E14.5 and sections 2 days later. While control cells migrate continuously toward the CP, the migration of neurons electroporated with shCntn1 was dramatically delayed. Time = hh:mm. **(D)** Statistical analysis of the rate of migration in control and Cntn1-KD cells 2 days after electroporation. *n* = 15–20 cells in 3 animals. ^∗∗∗^*p* < 0.001, student’s *t*-test. Data are shown as mean ± *SD*.

To investigate the identify of these electroporated cells at this stage, we performed immunostaining for neural progenitor markers Pax6 and Tbr2 as well as neuronal markers Tuj1 and NeuN (Figure 2). We found that, in both control and Cntn1 KD conditions, most of the GFP+ cells were negative to Pax6 and Tbr2 2 days after electroporation (Supplementary Figure 3). At day 4, the majority of GFP+ cells became Tuj1+ (Figure 2A), suggesting a neuronal lineage, although they were not positive to NeuN (Figure 2B), whose expression comes on later during development.

To overcome the possible lack of Cntn1 shRNA expression in GFP+ cells resulted from co-electoporation experiments, we electroporated E14.5 mouse brains with shCntn1-GFP construct, which expresses both shCntn1 and GFP. We found very similar delay in neuronal relocation to the CP, compared to control brains electroporated with shCtrl-GFP (Supplementary Figure 4). These results are consistent with our recent study using CRISPR/Cas9 to disrupt *Cntn1* in the mouse cortex (Lu et al., 2018).

To directly observe the neuronal migration defects, we performed time-lapse cell imaging in live brain slices. Brain slices were cultured under a fluorescent microscope 2 days after electroporation at E14.5. While control neurons extended a leading process and migrated away from the VZ at ∼0.8 μm/min, the migration rate of neurons electroporated with shCntn1 were dramatically decreased (Figures 2C,D). These results demonstrate that Cntn1 KD indeed leads to migration delay of neurons in the developing cortex.

### Abnormal Morphology and Processes of Cntn1-KD Neurons

**FIGURE 3:**
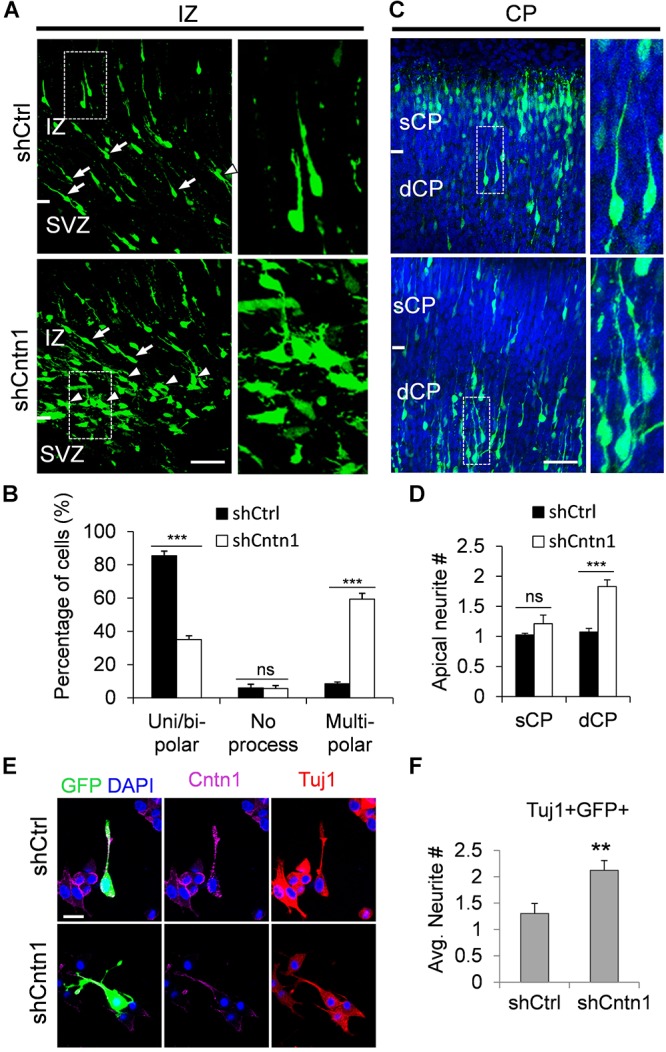
Abnormal process and cell morphologies in Cntn1-KD neurons. **(A)** In the IZ of control brains, most cells appeared uni/bipolar morphology (arrows and boxes). In brains electroporated with shCntn1, more multipolar cells were observed in the IZ (arrowheads). Bar = 100 μm. **(B)** Statistical analysis of the percentages of uni/bipolar, no process, and multipolar cells in the IZ. **(C)** In the CP of control brains electroporated with EGFP (green) and shCtrl, most cells in the CP presented a single leading process. In the deeper CP (dCP) of brains electroporated with shCntn1, cells possessed multiple leading processes (box). DAPI: blue. Bar = 100 μm. **(D)** Statistical analysis of average apical neurite number of cells in the sCP and the dCP. sCP, superficial CP; dCP, deeper CP. **(E)** Cultured cortical neurons transfected with control or Cntn1 shRNA (green) stained with neuronal marker Tuj1 (red) and DAPI (blue). shCntn1-transfected cells presents multiple processes. Bar = 10 μm. **(F)** Bar graph shows that cells transfected with shCntn1 presents more neurites in comparison with control cells. **(B,D,F)** ns: *p* > 0.05, ^∗∗^*p* < 0.01, ^∗∗∗^*p* < 0.001, one way ANOVA, Bonferroni *post hoc* correction. Data are shown as mean ± *SD*.

In order to investigate how Cntn1 KD results in altered neuronal migration and differentiation, we observed the morphology of electroporated cells in different regions of the developing brain (Figure 3). In the IZ, most cells in the control group presented uni/bipolar morphology 4 days after electroporation (85.5 ± 2.7%, *n* = 4 animals; Figure 3A); whereas significantly less cells were multipolar (8.5 ± 1.0%, *n* = 4 animals). In contrast, many cells in the Cntn1-KD group presented multipolar morphology (59.3 ± 3.5%, *n* = 4 animals; Figure 3A). Therefore, Cntn1-KD cells appeared to exhibit defects in the multipolar-to-bipolar transition in the SVZ (Figure 3B).

Interestingly, although some of the Cntn1-KD neurons were able to transit from multipolar to bipolar morphology, presented multiple leading processes (1.8 ± 0.11, *n* = 4 animals) with irregular morphology (Figures 3C,D), particularly in the dCP and IZ. In comparison, the control migrating neurons possessed only a single leading process (1.1 ± 0.06, *n* = 4 animals). The processes also appeared more curly and contained varicosities, suggesting defects in process morphogenesis. Neuronal cells electroporated with shCntn1-GFP also exhibited similar aberrant leading processes in the dCP (Supplementary Figure 4), further confirming effects of Cntn1 KD in the morphogenesis of migratory neurons.

To verify this phenomenon *in vitro*, we transfected E14.5 cortical neurons with control or Cntn1 shRNA and counted the average process of neurites 2 days later (Figure 3E). We found that the number of neurites of Cntn1-KD neurons was indeed increased compared to that of control cells (Figure 3F). Altogether, these results suggest that Cntn1 may play important roles in neuronal process formation and influence the multipolar-to-bipolar transition of newborn neurons during cortical development.

### Increased RhoA Activity by Cntn1 KD in the Developing Cortex

**FIGURE 4:**
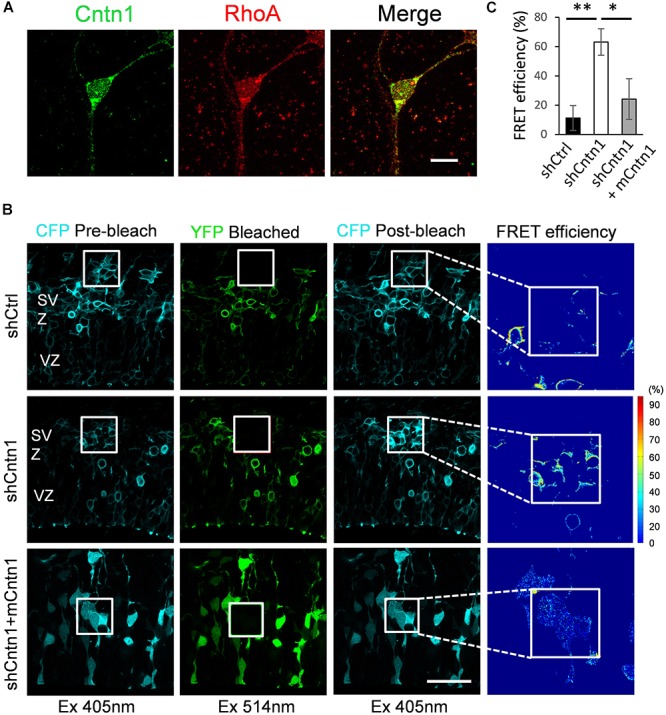
Increase in RhoA activity in cells transfected with Cntn1 shRNA. **(A)** Immunostaining of Cntn1 (green) and RhoA (red) in cultured cortical neurons. Cntn1 and RhoA are colocalized (appears yellow) in both the cell body and neurites. Bar = 10 μm. **(B)** RhoA activity measured by FRET analysis in neural progenitors expressing pRaichu-RhoA biosensor 2 days after *in utero* electroporation. CFP fluorescence was quenched by FRET to YFP (1st column). After bleaching of YFP in the designated areas (white boxes; 2nd column), CFP fluorescence was restored (3rd column). FRET efficiency was calculated based on the extent of the FRET quenching activity (4th column). RhoA in its active form leads to higher FRET efficiency. Cells co-transfected with shCntn1 exhibited higher FRET efficiency compared to that in control cells. The increase in RhoA activity by shCntn1 was reversed by re-introducing mCntn (bottom row). Bar = 50 μm. **(C)** Bar graph shows the FRET efficiency in brains electroporated with shCtrl, shCntn1 and shCntn1 + mCntn1. ^∗^*p* < 0.05, ^∗∗^*p* < 0.01, one way ANOVA, Bonferroni *post hoc* correction. Data are shown as mean ± *SD*.

To examine how Cntn1 delayed neuronal migration and affected neurite morphogenesis in the developing cortex, we searched potential downstream targets of Cntn1 in the literature. Previously, the small GTP-binding protein RhoA has been demonstrated to be involved in Cntn1-induced F-actin polymerization during cancer cell migration (Su et al., 2006). In addition, RhoA is expressed in the developing neocortex (Olenik et al., 1999) and regulates neuronal migration (Azzarelli et al., 2014a). To test whether Cntn1 KD could affect RhoA activities, we first examined whether Cntn1 and RhoA are colocalized in neurons by immunostaining and confocal microscopy (Figure 4A). We found that RhoA is highly colocalized with Cntn1 as puncta at the bottom of the soma and along the neurites of mouse cortical neurons cultured *in vitro*.

To further examine RhoA activity under the influence of Cntn1 knockdown *in vivo*, we measured RhoA activity in cortical neurons by FRET analysis. A FRET probe for RhoA (Yoshizaki et al., 2003; Nakamura et al., 2005; Pertz et al., 2006; Azzarelli et al., 2014b) was electroporated with Cntn1 shRNA or a shCtrl to the cortex at E14.5 followed by FRET analysis in brain slices 2 days later (Figures 4B,C). RhoA activity was detected in SVZ and lower IZ cells in brain slices. We found that the FRET efficiency, and hence RhoA activity, was significantly enhanced by Cntn1-KD (Figures 4B,C). Re-introduction of mCntn1 into the brain slices decreased the FRET efficiency back to the same level as control cells (Figures 4B,C). These data therefore indicate that Cntn1 inhibits RhoA activity in migrating cortical neurons during brain development.

### Rescue of Neuronal Migration and Morphology by RhoA Inhibition

**FIGURE 5:**
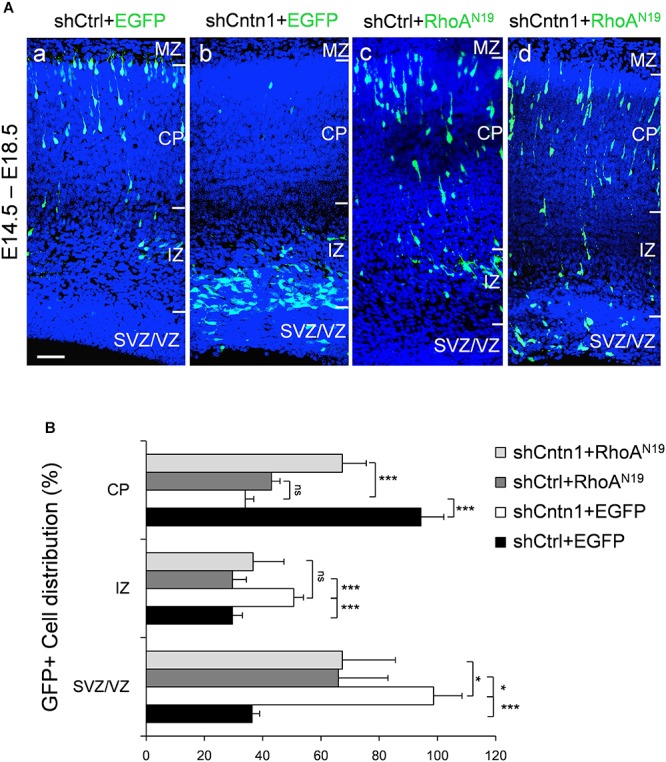
Expression of dominant-negative RhoA rescued migration defects resulting from Cntn1 KD. **(A)** Distribution of neurons in brain slices 4 days after co-electroporation of Cntn1 shRNA and dominant-negative RhoA (RhoA^N19^). **(a)** In the control brains electroporated with shCtrl along with EGFP (green), most cells have reached the CP. **(b)** Most cells expressing shCntn1 and EGFP are arrested in the IZ and SVZ, exhibiting a migration delay. **(c)** Expression of RhoA^N19^ alone showed some changes in cell distribution compared to the control brains in **(a)**. **(d)** Co-electroporation of RhoA^N19^ along with shCntn1 reversed the migration defects observed in **(b)**, producing cell distributions similar to those in control brains in **(a)**. Blue: DAPI. Bar = 50 μm. **(B)** Bar graph of cell distribution in the brain 4 days after electroporation of shRNAs with or without RhoA^N19^. ns, not significant. ^∗^*p* < 0.05, ^∗∗∗^*p* < 0.001, one way ANOVA, Bonferroni *post hoc* correction. Data are shown as mean ± *SD*.

To investigate whether Cntn1 indeed regulated neuronal migration through modulating RhoA activity *in vivo*, we co-expressed a dominant-negative form of RhoA, RhoA^N19^ (Olson et al., 1995), with Cntn1 shRNA to test whether RhoA^N19^ could rescue the migration defects caused by Cntn1 KD. Remarkably, expression of RhoA^N19^ in the developing neurons partially rescued the migration defect resulted from Cntn1 KD (Figure 5). Interestingly, expression of RhoA^N19^ alone caused some migration defects at this stage, consistent with previous reports (Xu et al., 2015).

**FIGURE 6:**
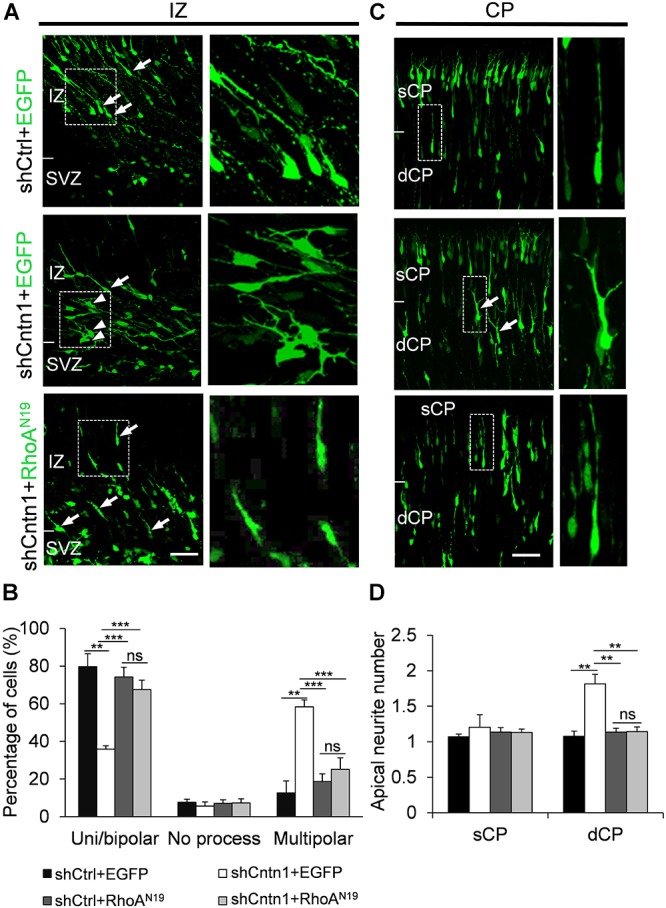
Expression of RhoA^N19^ rescued abnormal neurite morphology resulted from Cntn1 KD. **(A)** Cell morphologies in the IZ. Most control neurons exhibited a uni/bipolar morphology (arrows). Cntn1 KD increased the percentage of multipolar cells (arrowheads). Co-expression of RhoA^N19^ with Cntn1 shRNA increased the proportion of uni/bipolar cells (arrows). The Right panels represent enlarged images from boxes in the left panels. Bar = 50 μm. **(B)** Statistical analysis showing the apical neurite number of the cells with or without RhoA^N19^ co-expression in the sCP and dCP. **(C)** Cell morphologies in the CP. Control neurons expressing shCtrl and EGFP (green) normally possess a single leading process. Cntn1 KD induced branching in the leading process of migrating neurons (arrows). Co-expression of RhoA^N19^ and Cntn1 shRNA in the cell rescued the branching phenotype back to normal. The Right panels represent enlarged images from boxes in the left panels. **(D)** Statistical analysis showing the percentages of uni/bipolar, no process, and multipolar cells with or without RhoA^N19^ co-expression in the IZ. ns: *p* > 0.05, ^∗∗^*p* < 0.01, ^∗∗∗^*p* < 0.001, one way ANOVA, Bonferroni *post hoc* correction. Data are shown as mean ± *SD*.

In addition, we found that that expression of RhoA^N19^ restored the morphology of migrating cells (Figure 6). While Cntn1-KD cells had multiple branches in their leading process, cells co-electroporated with shCntn1 and RhoA^N19^ possessed a single leading process, exhibiting similar morphology as control migrating neurons. Moreover, in the IZ, brains co-electroporated with shCntn1 and RhoA^N19^ had increased percentages of uni/bipolar neurons compared to brains with sole shCntn1 electroporation (Figure 6). Altogether, these results suggest that Cntn1 regulates neuronal migration in the developing cortex through the RhoA pathway.

## Discussion

**FIGURE 7:**
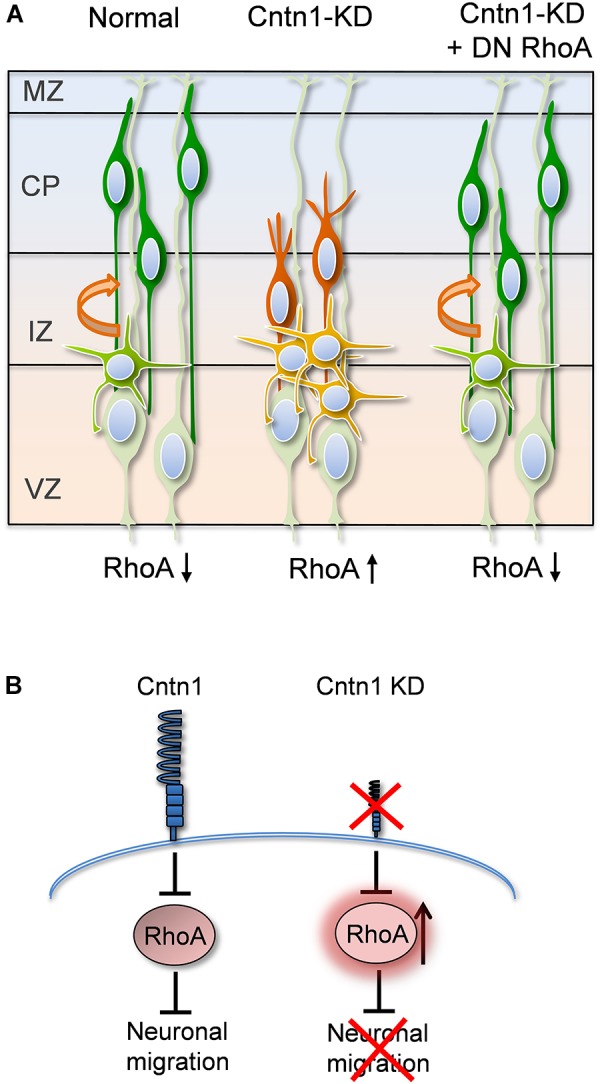
Proposed model for Cntn1 function in the neuronal migration during corticogenesis **(A)** In the normal brain, newborn neurons (blue) migrate from the VZ along the radial fiber of RGCs to the CP (Kriegstein and Noctor, 2004). Cntn1 dysfunction arrests neurons in the multipolar stage and caused branching of the leading process in the bipolar stage. These phenotypes lead to defects in neuronal migration. These defects can be rescued by inhibition of RhoA activities. **(B)** At the molecular level, our results suggest that Cntn1 regulates neurite morphologies and neuronal migration through inhibiting RhoA activity during cortical development. Loss of Cntn1 increases RhoA activity causing neuronal migration defects.

In the current study, we found that Cntn1 is essential for the radial neuronal migration and final localization of neurons in the developing mouse neocortex. Cntn1 KD by *in utero* electroporation significantly delayed radial migration (Figures 1, 2). Cntn1 KD also perturbed the multipolar-to-bipolar transition of migrating neurons and led to abnormal branching of the leading process (Figure 3). Moreover, Cntn1-KD neurons exhibited higher activity of RhoA, a Rho-GTPase critical for actin cytoskeletal reorganization (Figure 4). Overexpression of dominant-negative RhoA successfully rescued defects in neuronal migration and morphogenesis resulting from Cntn1 KD (Figures 5, 6). These results suggest that Cntn1 may regulate neuronal migration through modulating RhoA activity. Therefore, we propose a novel model of neuronal migration during corticogenesis: through regulating RhoA activity, Cntn1 facilitates multipolar-to-bipolar transition and then maintains a normal leading process. These roles of Cntn1 in turn promote neuronal migration in the developing cerebral cortex (Figure 7).

### Role of Cntn1 in Adhesion and Neuronal Migration

Consistent with our previous genetic screen (Lu et al., 2018), we showed that Cntn1 KD delayed the migration of cortical neurons during brain development, resulting in mis-positioning of Cntn1-deficient neurons in mice. Some of these ectopically cells mis-positioned under cortical layer II-IV after birth (Lu et al., 2018). Moreover, the Cntn1-KD neurons exhibited aberrant process morphology during neuronal migration. Since Cntn1 is involved in regulating neural cell adhesion during cerebellar development, axonal extension, and myelination (Berglund et al., 1999; Faivre-Sarrailh et al., 1999; Falk et al., 2002; Karagogeos, 2003), we suspected that Cntn1 deficiency in cortical neurons may affect their adhesion to radial fibers and/or neurites of subplate neurons (Ohtaka-Maruyama et al., 2018), thus hindering the relocation of neural precursors to the developing cerebral cortex (Figure 3).

The binding counterparts of Cntn1 in the neuropile or on the surface of radial glial fibers or subplate neurites remain unclear. In previous studies, Cntn1 has been shown to associate with other cell membrane proteins involved in various signal transduction pathways. For example, Cntn1 interacts with the α-isoform of receptor protein tyrosine phosphatase (RPTPα) (Zeng et al., 1999) to transduce extracellular signals to Fyn kinase, a member of the Src kinase family that promotes neurite outgrowth and attraction (Umemori et al., 1994; Liu et al., 2004). Cntn1 also interacts with the RPTPβ (Revest et al., 1999), which binds to different kinds of cell adhesion molecules and components of the extracellular matrix, including NCAM and pleiotrophin. Importantly, both RPTPα and RPTPβ interact with and regulate the tyrosine phosphorylation of catenins, proteins that are essential to many physiologic events, such as cell migration, adhesion, and transformation (Beltran and Bixby, 2003). Indeed, RPTPβ is localized along radial glia fibers as well as on neurons and has been shown to be involved in neuronal migration during cortical development (Canoll et al., 1993; Maeda et al., 1995; Maeda and Noda, 1998; Lamprianou et al., 2011). Therefore, future studies for Cntn1-ligand interaction would be beneficial to conduct as they could shed light on neuron-glia interaction and regulation during cortical neuron migration.

### Cntn1 Regulates Neuronal Migration via Modulating RhoA GTPase Activity

Another interesting finding in our study is that Cntn1 KD led to a higher RhoA activity in migrating neurons (Figure 4). More importantly, RhoA inhibition by DN-RhoA (RhoA^N19^) expression successfully rescued the migration defects caused by Cntn1-KD (Figure 5). These results suggest that Cntn1 mediates neuronal migration not only through adhesion but also through inhibition of RhoA, a major regulator of the cytoskeleton. RhoA belongs to the small Rho GTPase family, some of which have been shown to play essential functions in cortical development (Azzarelli et al., 2014a). The most extensively studied members of the Rho family include RhoA, Rac1, and Cdc42, which are best characterized by their effects on the cytoskeleton and cell adhesion. On one hand, RhoA is down regulated to promote radial migration of pyramidal neurons during cortical development (Azzarelli et al., 2014a). Inhibition of RhoA is also required for multipolar-to-bipolar transition (Pacary et al., 2011). Higher activities of RhoA in neurons lead to neuronal migration delay and multipolar dendritic morphologies (Threadgill et al., 1997; Tang et al., 2014). On the other hand, expression of dominant negative RhoA delays neuronal migration (Xu et al., 2015). Interestingly, RhoA deficient mice exhibited neuronal migration defects in a RGC-dependent but neuron-independent manner (Cappello et al., 2012). Therefore, appropriate RhoA regulation at different stage and cell type is crucial to the complex process of neuronal migration.

The Rho GTPases regulate many aspects of actin cytoskeletal dynamics, including the formation of stress fibers, filopodia, and lamellipodia (Cappello, 2013). Among them, RhoA is responsible for the formation of stress fibers, and its activity can affect the assembly, disassembly, or reorganization of the actin cytoskeleton (Pellegrin and Mellor, 2007; Heng et al., 2010). Here we propose that RhoA inhibition by Cntn1 may decrease stress fiber and actin filament formation, thus modulating process dynamics and promoting neuronal migration.

### Contactin and Human Neurological/Psychiatric Disorders

A previous report described a *CNTN1* mutation that caused a lethal form of congenital myopathy in 4 members of a large consanguineous Egyptian family patients, possibly due to a loss of contactin-1 in the neuromuscular junction (Compton et al., 2008). These infants were born prematurely and had an absence of spontaneous movement. Although abnormality in the brain was not reported, our findings warrant more detailed investigation of the morphological and histological changes in the brain of patients with *CNTN1* mutations. It has also been reported that mutations in human *CNTNAP2* gene, which encodes a contactin-2 binding protein, is associated with seizures, mental retardation, schizophrenia and autism spectrum disorder (Strauss et al., 2006; Alarcon et al., 2008; Arking et al., 2008; Bakkaloglu et al., 2008; Friedman et al., 2008). Interestingly, variations of other human contactin genes, CNTN3-6, have been found to be associated with autism spectrum disorder (Morrow et al., 2008; Burbach and van der Zwaag, 2009; Roohi et al., 2009; Stankiewicz and Lupski, 2010). Recently, we also found somatic mutations in *CNTN1* in the brain lesion of patients with focal cortical dysplasia (FCD) (Lu et al., 2018). Although the exact impact of these genetic variations remains elusive, it is evident contactins play a vital role in human neural development.

In summary, our findings suggest that the Cntn1 controls the precise morphogenesis, migration, and final localization of cortical neurons. Cntn1 regulates neuronal migration through inhibiting RhoA activity, which in turn modulates the actin cytoskeleton during cortical development. Our results lead to a better understanding of cell adhesion molecules on the neuronal migration pathway, which may unveil specific mechanisms underlying a wide spectrum of neural developmental disorders.

## Author Contributions

Y-AC and I-LL designed and performed the experiments, analyzed the data, and contributed to the manuscript. J-WT designed and oversaw the study and revised the manuscript. All authors read and approved the final manuscript.

## Conflict of Interest Statement

The authors declare that the research was conducted in the absence of any commercial or financial relationships that could be construed as a potential conflict of interest.

## Abbreviations

CP: cortical plate
dCP: deeper CP
E: embryonic day
EGFP: enhanced green fluorescent protein
IZ: inter mediate zone
KD: knockdown
P: postnatal day
RGC: radial glial cell
RPTP: receptor protein tyrosine phosphatase
sCP: superficial CP
shCntn1: Cntn1 shRNA
shCtrl: control shRNA
shRNA: short hairpin RNA
SVZ: subventricular zone
VZ: ventricular zone
