# Supplementary material for: Contactin-1/F3 Regulates Neuronal Migration and Morphogenesis Through Modulating RhoA Activity
Source: Front Mol Neurosci. 2018 Nov 20;11:422. doi: 10.3389/fnmol.2018.00422 (PMC6255823; doi:10.3389/fnmol.2018.00422)
Supplement: Supplementary file 1 [file Data_Sheet_1.PDF]

Supplementary Material

Contactin-1/F3 Regulates Neuronal Migration and Morphogenesis through Modulating RhoA Activity

Yi-An Chen, I-Ling Lu, and Jin-Wu Tsai*

*** Correspondence:** Corresponding Author: tsaijw@ym.edu.tw

1 Supplementary Figures and Tables

1.1 Supplementary Figures

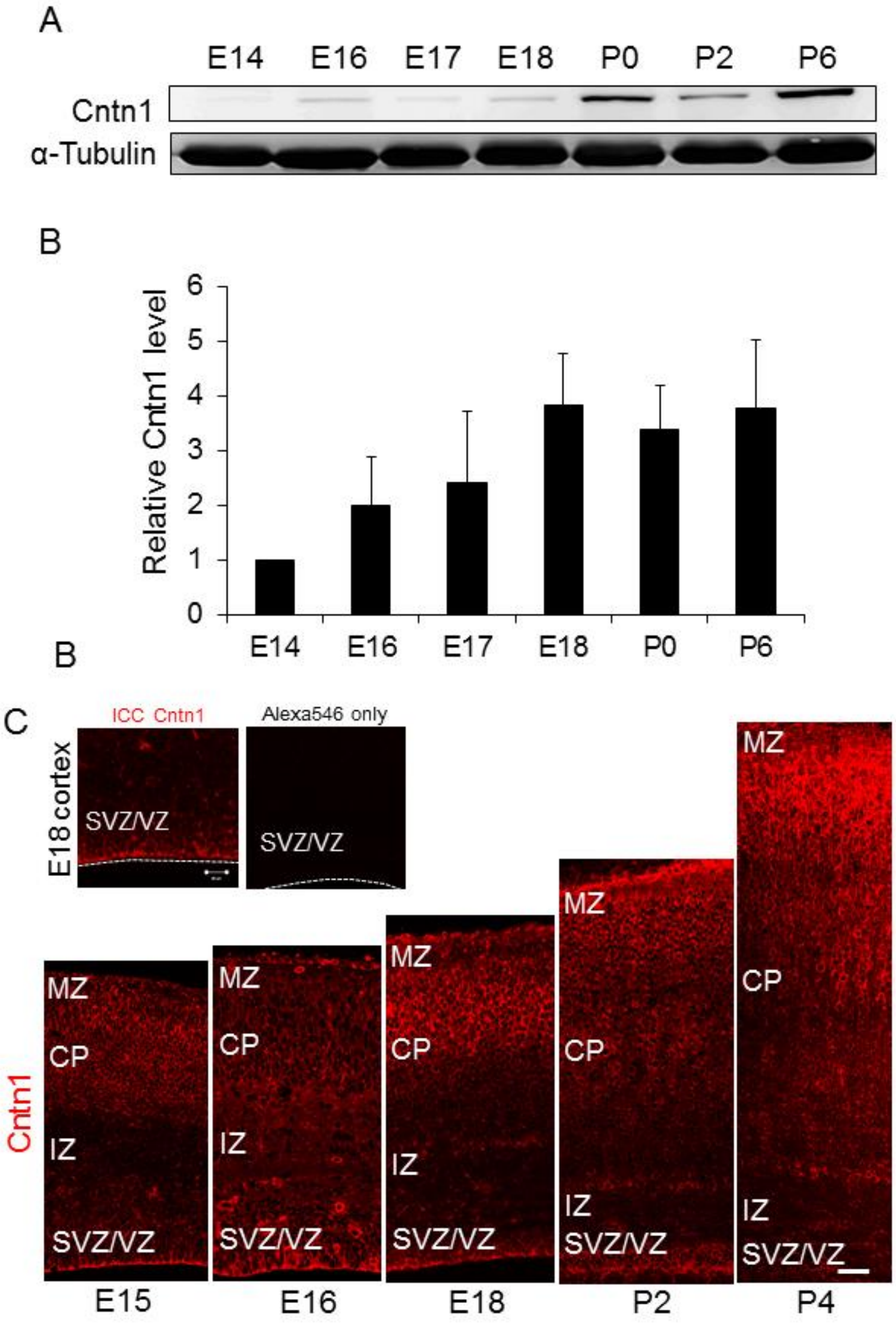

Supplementary Figure 1. Expression of Cntn1 in the developing mouse cortex. (A) Western blot showing Cntn1 expression in cortical lysates at different developmental stages from E14 to P6. (B) Bar graph demonstrating upregulation of Cntn1 expression in E14 to E18 in addition to neonatal stages. n = 3 animals. Data are shown as mean ± SD. (C) Immunohistochemistry staining of Cntn1 expression (red) at different developmental stages. At E16, Cntn1 was modestly expressed in the SVZ/VZ. At E18, expression was robust in the CP, and in the scatter cells deeper in the CP and IZ. By P4, robust expression was detected throughout most of the CP. No immunoreacitivity was detected using secondary antibody only. Bar = 50μm.

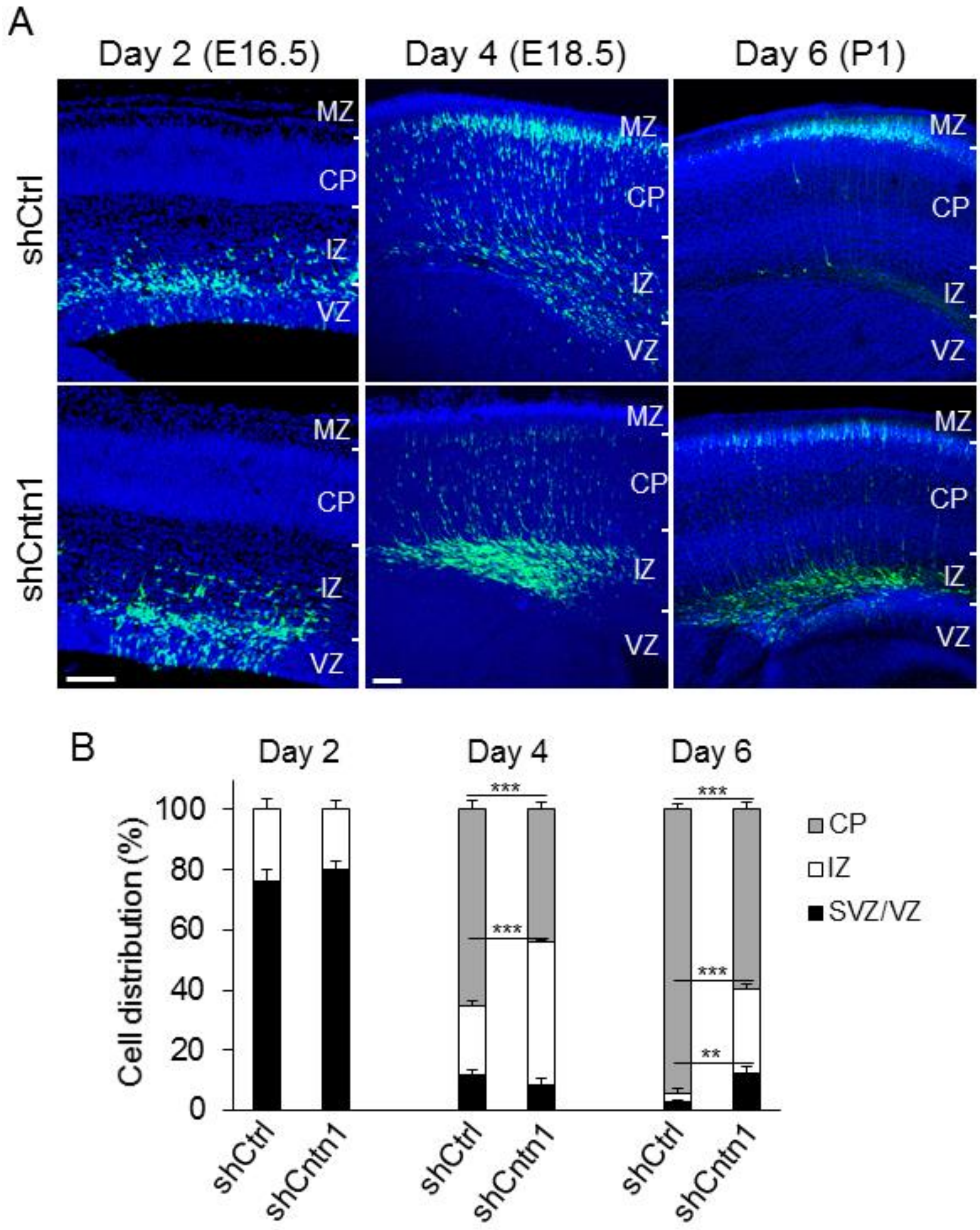

Supplementary Figure 2. Cell distributions in the brains electroporated with shCntn1. (A) Distribution of cells electroporated with shCntn1 or shCtrl with EGFP (green) at E14.5 in the developing mouse cortex for 2, 4, and 6 days. Most electroporatedcells in both groups were located in the VZ at day 2. In control brains electroporated with shCtrl, cells migrated from the VZ to the CP in the subsequent days (upper panel). In brains electroporated with shCntn1 many cells were restricted to the VZ and IZ at day 4 (n = 6) and day 6 (n = 4) (lower panel). Blue: DAPI. Bar = 100μm. (B) Bar graph of the cell distribution at different days after electroporation. * : p < 0.05, ** : p < 0.01, *** : p < 0.001, student's t-test. Data are shown as mean ± SD.

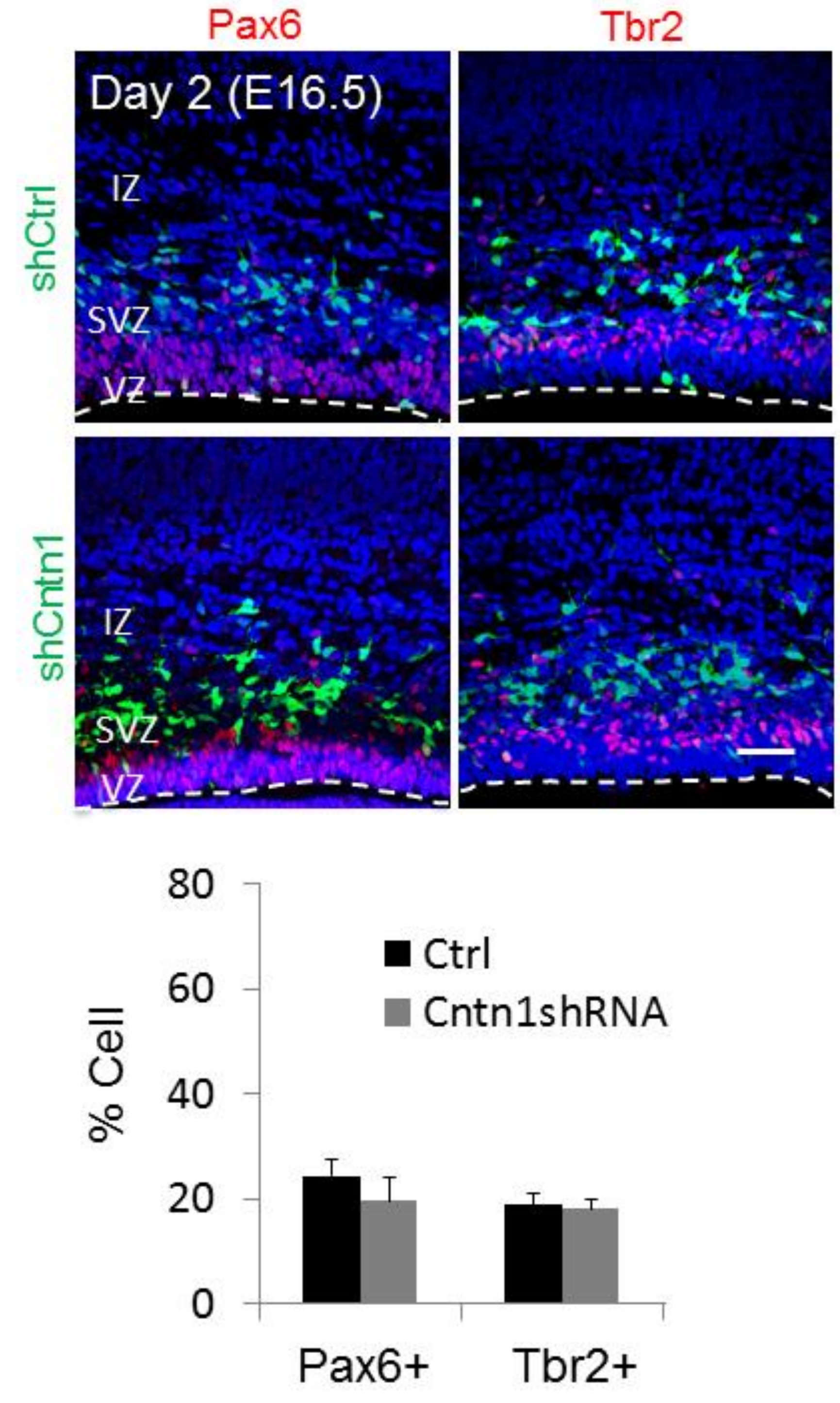

Supplementary Figure 3. Immunostaining of progenitor cell markers Pax6 (for RGCs) and Tbr2 (for intermediate progenitors) in brains electroporated with control and Cntn1 shRNA. Brains electroporated with shCtrl and shCntn1 (green) at E14.5 were stained with these antibodies (red) 2 days after electroporation. Most cells arrested has moved to the SVZ and IZ and did not express these markers. Bar = 50µm.

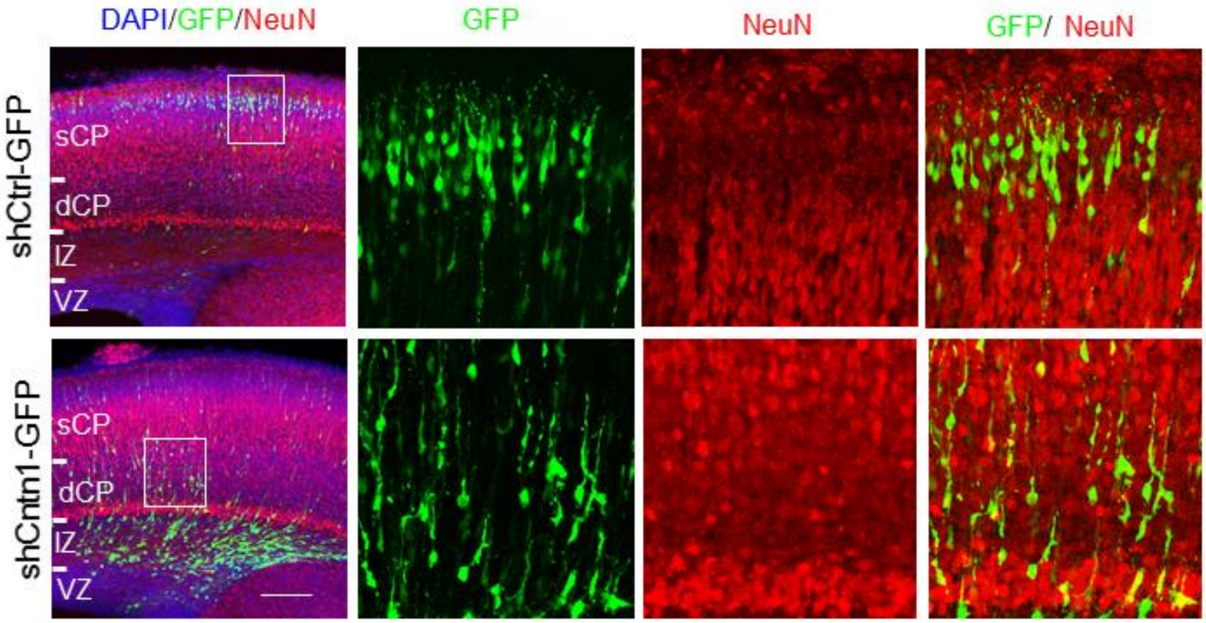

Supplementary Figure 4. Immunostaining of neuronal marker NeuN in brains electroporated with control or Cntn1 shRNA. Brains electroporated with shCtrl-GFP or shCntn1-GFP, which expresses shRNA and GFP (green) were stained with NeuN antibody (red). Neurons electroporated with shCntn1-GFP exhibited aberrant leading processes with branched and curly morphology in the deep CP. Brain slices were stained with DAPI (blue). The 3 panels on the right represent enlarged images from the boxes on left. Bar = 100μm.
